# Galectin-9 expression correlates with therapeutic effect in rheumatoid arthritis

**DOI:** 10.1038/s41598-021-85152-2

**Published:** 2021-03-10

**Authors:** Jiao Sun, Yameng Sui, Yunqing Wang, Lijun Song, Dong Li, Guosheng Li, Jianwei Liu, Qiang Shu

**Affiliations:** 1 Department of Rheumatology, Qilu Hospital, Cheeloo College of Medicine, Shandong University, Jinan, 250012 Shandong China; 2 Department of Nephrology and Immunology, Shandong Provincial Third Hospital, Shandong University, Jinan, 250031 Shandong China; 3 Department of Rheumatology and Immunology, Yantai Mountain Hospital, Yantai, 264001 Shandong China; 4 Department of Rheumatology and Immunology, Guangdong Second Provincial General Hospital, GuangzhouGuangdong, 510317 China; 5 Shenzhen Research Institute of Shandong University, Guangdong, 518057 Shenzhen China; 6 Cryomedicine Laboratory, Qilu Hospital, Cheeloo College of Medicine, Shandong University, Jinan, 250012 Shandong China; 7 Department of Hematology, Qilu Hospital, Cheeloo College of Medicine, Shandong University, Jinan, 250012 Shandong China

**Keywords:** Diseases, Rheumatology

## Abstract

Galectin-9 (Gal-9) is a multifunctional immunomodulatory factor highly expressed in RA. This study aimed to investigate the expression of Gal-9 and its correlation with disease activity and therapeutic response in RA patients. Active RA patients were enrolled and treated with tacrolimus (TAC) alone or in combination therapy for 12 weeks in a prospective cohort study. Clinical and immunological parameters were recorded at baseline and week 12. We measured Gal-9 expression in different T cell subsets and in plasma. The disease activity of RA patients decreased after treatment. At baseline, the Gal-9 expression percentage was higher in the group with severe disease than in mild or moderate groups. After treatment, the Gal-9 expression in CD3^+^, CD4^+^, CD8^+^ and CD4^-^CD8^−^ cell subsets decreased, as well as Gal-9 mean fluorescence intensity in CD3^+^, CD4^+^ and CD8^+^ T cells. Similarly, plasma Gal-9 levels were lower at week 12 than at baseline. Good responders showed significantly lower Gal-9 expression on CD3^+^ and CD4^+^ T cell subsets and lower plasma Gal-9 levels than poor responders. Gal-9 expression positively correlates with disease activity in RA patients. Gal-9 can be regarded as a new biomarker for evaluating RA activity and therapeutic effect, including TAC.

## Introduction

Rheumatoid arthritis (RA) is a common chronic autoimmune disease characterized by infiltration of inflammatory cells, active angiogenesis, and high fibroblast proliferation in the synovium^1^. The activation of T cells and related cytokines such as interleukin (IL)-6 and tumor necrosis factor (TNF)-α are involved in cellular immune responses during RA^2,3^.

Tacrolimus (TAC) is a calcineurin inhibitor used as immunosuppressor in rheumatic disease. TAC regulates the activity of T cells and inhibits the production of inflammatory cytokines such as TNF-α and interferon (IFN)^4^. Calcium-dependent phosphatase activates the nuclear factor of activated T cells transcription factor^5^. TAC can inhabit osteoclast formation through targeting the nuclear factor of activated T cells in RA^6^. Thus, TAC works as conventional disease-modifying anti-rheumatic drug (cDMARD) to reduce the systemic inflammatory response in patients with refractory RA^7^.

Galectin-9 (Gal-9) is a multifunctional member of the galectin family expressed in various cell types and involved in cell proliferation, differentiation, inflammation, tumor and immune cell formation^8^. Gal-9 and its receptor, T cell immunoglobulin domain and mucin domain-3 (Tim-3), are able to induce apoptosis of T cells in the pathogenesis of RA by regulating immune responses by T helper type 1 (Th1) and T helper type 17 (Th17) cells^9–11^. Moreover, Gal-9 promotes the differentiation of regulatory T cells (Tregs) to induce activated T cell apoptosis in tumor angiogenesis and immune escape^12^. On the other hand, Gal-9 synergizes with toll-like receptor (TLR) signaling pathways to promote Th1-mediated innate immune responses^13^.

Endogenous Gal-9 plays a pro-inflammatory role by suppressing apoptosis in human RA synovial fibroblasts^14^. Our previous studies showed that Gal-9 levels in peripheral blood mononuclear cells (PBMCs) and plasma are higher in RA patients than in healthy controls, and plasma Gal-9 level positively correlates with disease activity indexes in RA patients^15^. Whether Gal-9 plays a role in progression or remission of RA is still controversial. Therefore, Gal-9 may have undiscovered complex and diverse immune regulation and angiogenesis functions.

In this work, we aimed to verify the relationships among Gal-9, inflammatory cytokines and disease activity of RA, as well as analyze dynamic changes in Gal-9 expression in T cell subsets of PBMCs and in plasma. A prospective cohort study of patients with active RA treated with cDMARDs, including TAC, were performed.

## Results

### Clinical characteristics and disease activity at baseline and after treatment

**Figure 1:**
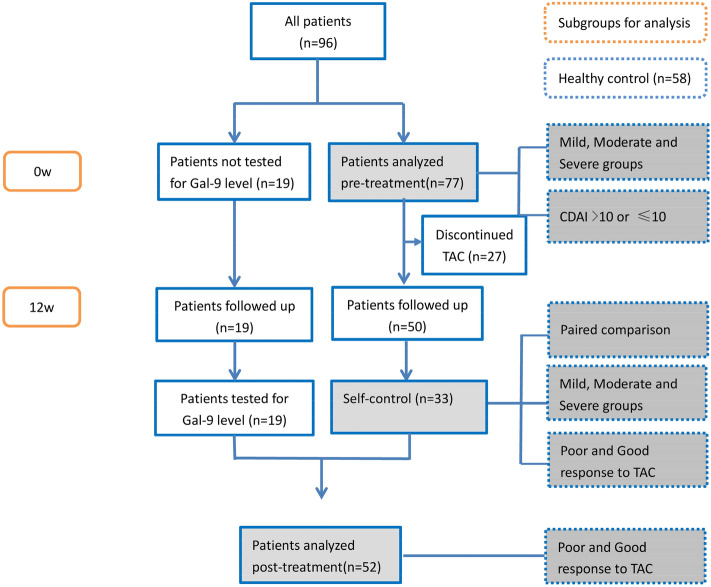
Flowchart of patient inclusion in the study. CDAI: Clinical disease activity index; TAC: Tacrolimus.

The flowchart of the study was shown in Fig. 1. Baseline clinical and demographic characteristics are presented in Table 1. Among the 77 patients with baseline Gal-9 data, there were 14 males and 63 females, with an average age of 52.0 ± 13.8 years. The average course of RA disease was 7.12 ± 6.84 years, and the average DAS28 score was 4.26 ± 1.3. There were 61 (79.2%) patients with moderate and severe disease activity. The clinical characteristics of 52 post-treatment patients and 33 RA patients with blood samples available before and after treatment are compared. A total of 58 healthy control(controls) were enrolled in this study, including 16 males and 42 females, with an average age of 47.86 ± 9.64.

**Table 1:** Patients demographic characteristics both at baseline and after 12 weeks of treatment.

	All patients (*N* = 96)	Pre-treatment (*n* = 77)	Post-treatment (*n* = 52)	Pre- and post-treatment (*n* = 33)
Female, *n*(%)	79 (82.3)	63(81.8)	45(86.5)	29(87.9)
Age (years)	51.1 ± 14.1	51.97 ± 13.78	49.88 ± 14.41	51.42 ± 13.91
Disease duration (years)	7.06 ± 7.14	7.12 ± 6.84	6.25 ± 6.92	5.92 ± 6.02
ESR(mm/h)	51.58 ± 29.47	51.27 ± 29.04	53.87 ± 29.06	56.52 ± 27.72
CRP (mg/L)	37.36 ± 37.20	40.21 ± 39.93	34.3 ± 34.76	39.18 ± 40.43
T28	7.40 ± 6.59	7.13 ± 6.28	7.13 ± 6.72	5.85 ± 4.7
SW28	5.36 ± 5.36	5.43 ± 5.31	4.96 ± 5.32	4.42 ± 4.26
PGA	5.59 ± 1.68	5.55 ± 1.63	5.63 ± 1.67	5.61 ± 1.46
HAQ	0.87 ± 0.57	0.88 ± 0.57	0.83 ± 0.55	0.76 ± 0.53
SDAI	60.01 ± 42.99	63.63 ± 46.4	57.26 ± 38.47	21.18 ± 9.71
CDAI	23.65 ± 13.66	23.43 ± 13.3	22.96 ± 13.47	60.37 ± 44.99
DAS28-CRP	4.20 ± 1.29	4.26 ± 1.3	4.08 ± 1.23	4.1 ± 1.2
**Tacrolimus treatment, n(%)**				
TAC	1 (1.04)	1(1.3)	1(1.9)	1(3.0)
TAC + M	15 (15.6)	14(18.2)	6(11.5)	5(15.2)
TAC + P	37(38.5)	26(33.8)	21(40.4)	10(30.3)
TAC + M + P	38(39.6)	31(40.3)	24(46.1)	17(51.5)
UK	5 (5.2)	5(6.5)	-	-

ESR: erythrocyte sedimentation rate, CRP: C-reactive protein, T28: tender joints of 28 counted, SW28: swollen joints of 28 counted, PGA: patient global assessment, HAQ: health assessment questionnaire, SDAI: simplified disease activity index, CDAI: clinical disease activity index, DAS28: disease activity score in 28 joints, TAC: tacrolimus, M: methotrexate, P: prednisone, UK: uncertain drugs combined with tacrolimus. Values are shown as mean ± standard deviation or n (%).

There were no significant differences in baseline demographic characteristics (gender, age, disease course) among the severe, moderate and mild groups of RA patients. The disease activity of patients was no difference between the Poor Response group (PR) and Good Response group (GR) at baseline. The patients who withdrew the study showed no differences from those finished the trial in baseline demographic characteristics.

We found that the doses of TAC, MTX and Pred was no statistical difference among the Mild, Moderate and Severe disease activity groups at both baseline and 12w. The blood concentration of TAC at 3 week time point were also no statistical difference among these three groups (Supplementary Table S1 online).

In this study, 19 cases in baseline and 44 cases after treatment were lost of blood samples, who used to bring a local laboratory test report for follow-up and lack of all Gal-9 value. There were 27 patients discontinued 12 weeks TAC treatment, including invalid (n = 2), stopping the drug by herself (n = 2), intolerance (n = 1), economic reasons (n = 8), infection (n = 4), unexplained reason (n = 1), lost to follow-up (n = 9).

### Gal-9 expression in 77 RA patients at baseline

**Figure 2:**
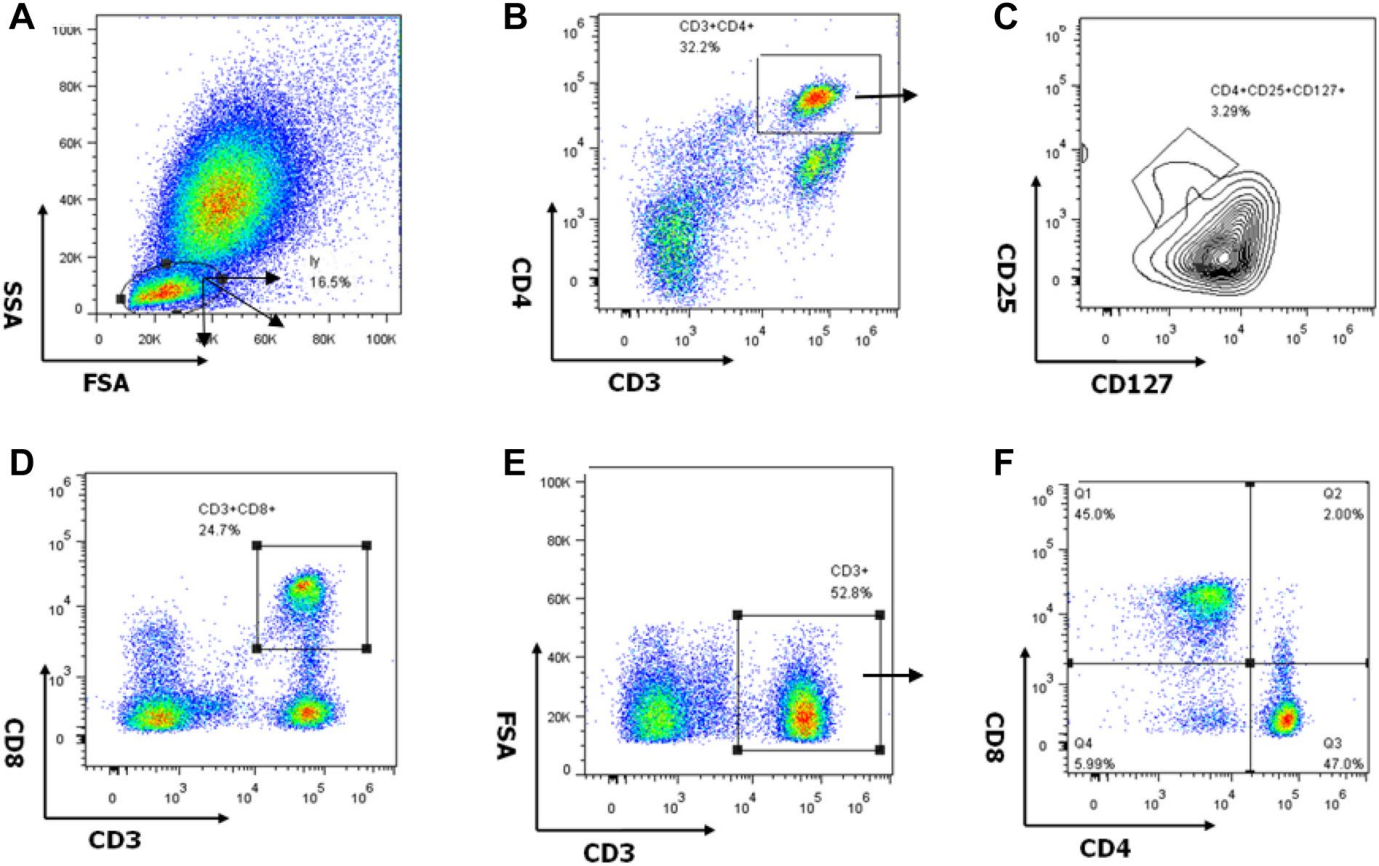
T cell subsets in peripheral blood mononuclear cells (PBMCs) of one rheumatoid arthritis patient showing the gating strategy used for flow cytometry analysis. (**A**) Lymphocytes in ungated PBMCs. (**B**) CD3^+^ CD4^+^ T cell subsets in lymphocytes. (**C**) CD25^+^ CD127^low^ (regulatory T cells, Tregs) in CD4^+^ T cell subsets. (**D**) CD3^+^ CD8^+^ T cell subsets in lymphocytes. (**E**) CD3^+^ T cell subsets in lymphocytes. (**F**) CD4^−^CD8^−^ (double-negative) T cell subsets in CD3^+^ T cells.

The gating strategy for different cell subsets is shown in Fig. 2. CD3^+^ cells were considered T lymphocytes, including the following subsets: CD4^+^ (CD3^+^CD4^+^CD8^−^), CD8^+^(CD3^+^CD4^−^CD8^+^), double-negative(CD3^+^CD4^−^CD8^−^), or Treg (CD4^+^CD25^+^CD127^low^).

**Figure 3:**
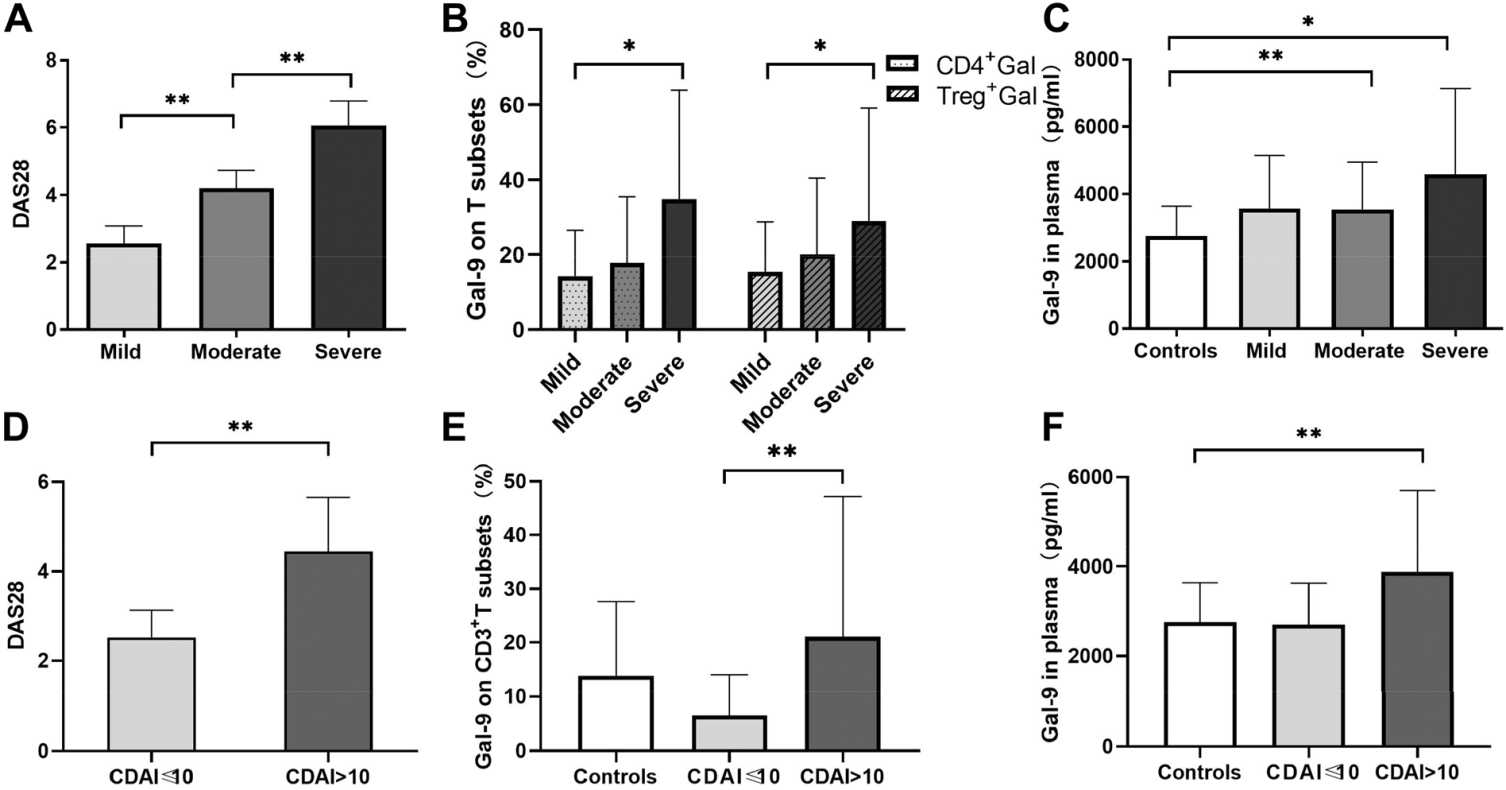
Disease activity and galectin-9 (Gal-9) levels in T cell subsets and plasma at baseline in different groups among 77 rheumatoid arthritis (RA) patients. Patients were divided into a mild group (Mi, n = 16), moderate group (Mo, n = 44) and severe group (Se, n = 17). Results were also compared with those of controls. (**A**) Groups according to disease activity score 28 (DAS28). (**B**) Gal-9 expression in CD4 + and regulatory (Treg) T cell subsets. (**C**) Gal-9 expression in plasma in different RA groups and controls (n = 58). (**D**–**F**) Differences in (**D**) DAS28, (**E**) Gal-9 expression in CD3 + T cell subsets, and (**F**) Gal-9 expression in plasma between groups with clinical disease activity index (CDAI) ≤ 10 (n = 7) or > 10 (n = 70). Comparisons between two groups were assessed using an independent-samples t-test, while comparisons among three groups were assessed using one-way ANOVA. *P < 0.05, **P < 0.01.

The expression of Gal-9 in the PBMCs of the severe group was higher than in the moderate and mild groups. The DAS28 score of the severe group was higher than that of the moderate (Fig. 3A). Gal-9 expression in CD4^+^ T and Treg cell subsets of severe patients was higher than that of mild patients (Fig. 3B). The percentages of CD4^+^ T and Treg cells in PBMCs did not differ significantly among the three groups.

The expression of Gal-9 in the plasma of 77 RA patients was higher than in the control group. High and moderate disease activity groups of patients had higher plasma levels of Gal-9 than controls (*P* = 0.015 in severe group, *P* = 0.003 in moderate group; Fig. 3C). In addition, plasma concentrations of VEGF (56 ± 123 *vs* 594 ± 810 pg/ml), TNF-α (13.71 ± 29.15 *vs* 5.46 ± 2.41 pg/ml) and IL-6 (14.49 ± 23.42 *vs* 0.9 ± 0.74 pg/ml) were significantly higher in RA patients than in healthy controls.

RA patients with high CDAI scores had higher value of DAS28 (*P* = 0.001; Fig. 3D), and also expressed higher Gal-9 levels. The percentage of CD3^+^ T cells expressing Gal-9 in PBMCs was higher in the high CDAI group than in the low CDAI group (*P* = 0.003; Fig. 3E). Plasma Gal-9 levels were higher in the high CDAI group than in controls (*P* = 0.000), while no significant difference was shown between the two CDAI groups (Figs. 3F).

### Gal-9 expression in 52 patients at week 12 post-treatment

After TAC-based treatment for 12 weeks, 30 of 52 RA patients showed good response, while 22 showed poor response. Good responders received TAC + M (n = 4, 13.3%), TAC + P (n = 14, 46.7%), or TAC + M + P (n = 12, 40.0%). Among the poor responders, one patient received only TAC (4.6%), two received TAC + M (9.1%), seven received TAC + P (31.8%), and 12 received TAC + M + P (54.6%).

**Figure 4:**
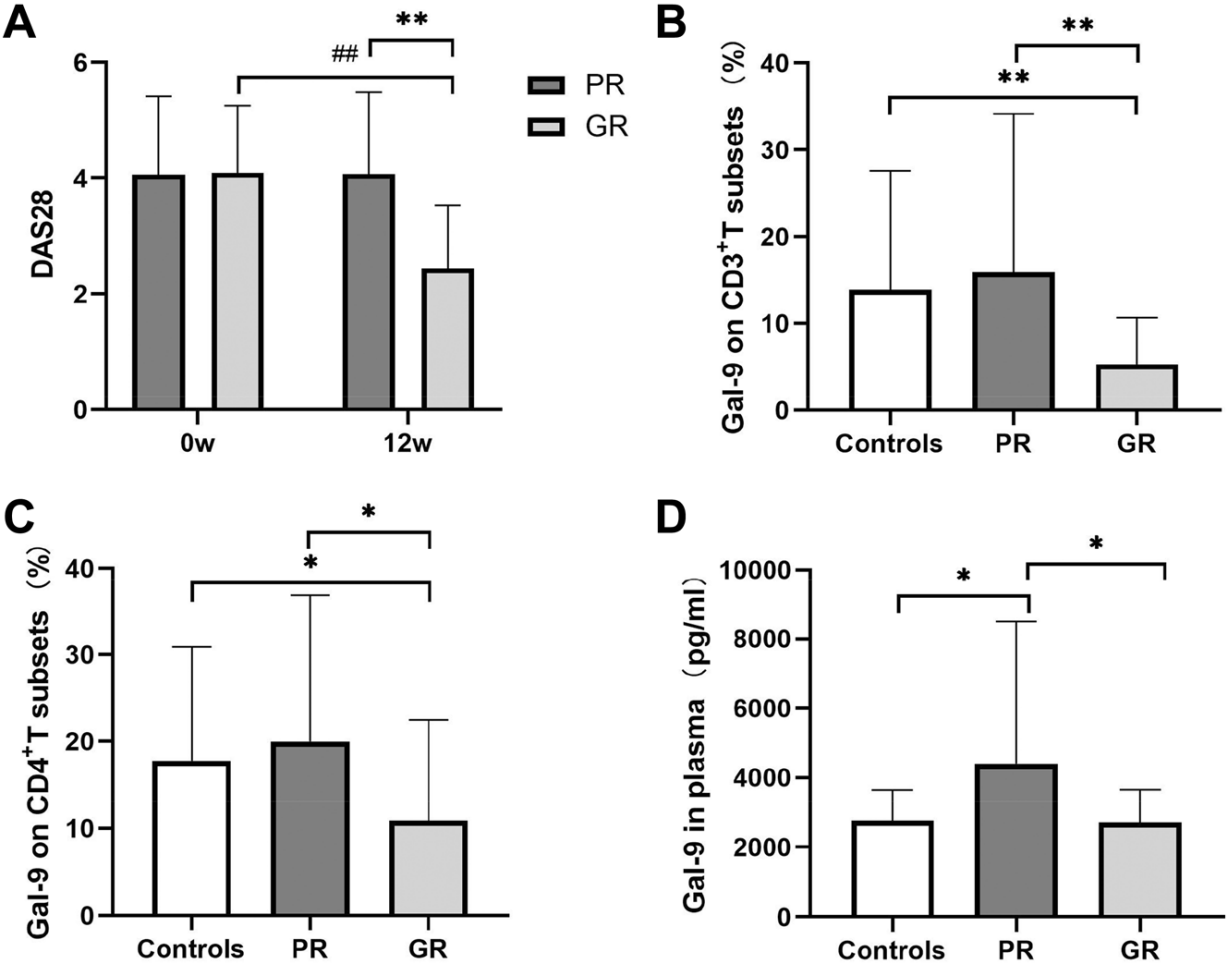
Disease activity and galectin 9 (Gal-9) levels in tacrolimus (TAC) treatment response groups (n = 52) and controls (n = 58). PR indicates poor response to TAC (n = 22), and GR indicates good response (n = 30). (**A**) Disease activity score 28 (DAS28). (**B**) Percentage of CD3^+^ T cells expressing Gal-9. (**C**) Percentage of CD4^+^ T cells expressing Gal-9. (**D**) Gal-9 expression in plasma. Significant differences between different groups at the same time point are marked as *(P < 0.05) or **(P < 0.01). Significant differences between baseline and 12 weeks within the same group are marked as # (P < 0.05) or ## (P < 0.01).

The good response group (1.97 ± 0.83) showed lower DAS28 score than the poor response group at 12 weeks (3.81 ± 1.06, P < 0.01; Fig. 4A), as well as lower Gal-9 expression in CD3^+^ and CD4^+^ T cell subsets (*P* = 0.020, *P* = 0.030, respectively; Fig. 4B–C). Good responders also showed significantly lower plasma Gal-9 levels at 12 weeks (2672 ± 930 *vs* 4377 ± 3989 pg/ml, *P* = 0.05; Fig. 4D). Together, the results indicate that low plasma Gal-9 expression levels and DAS28 score were performed in good response group.

### Decrease in Gal-9 expression in PBMCs and plasma after TAC treatment in 33 patients

**Figure 5:**
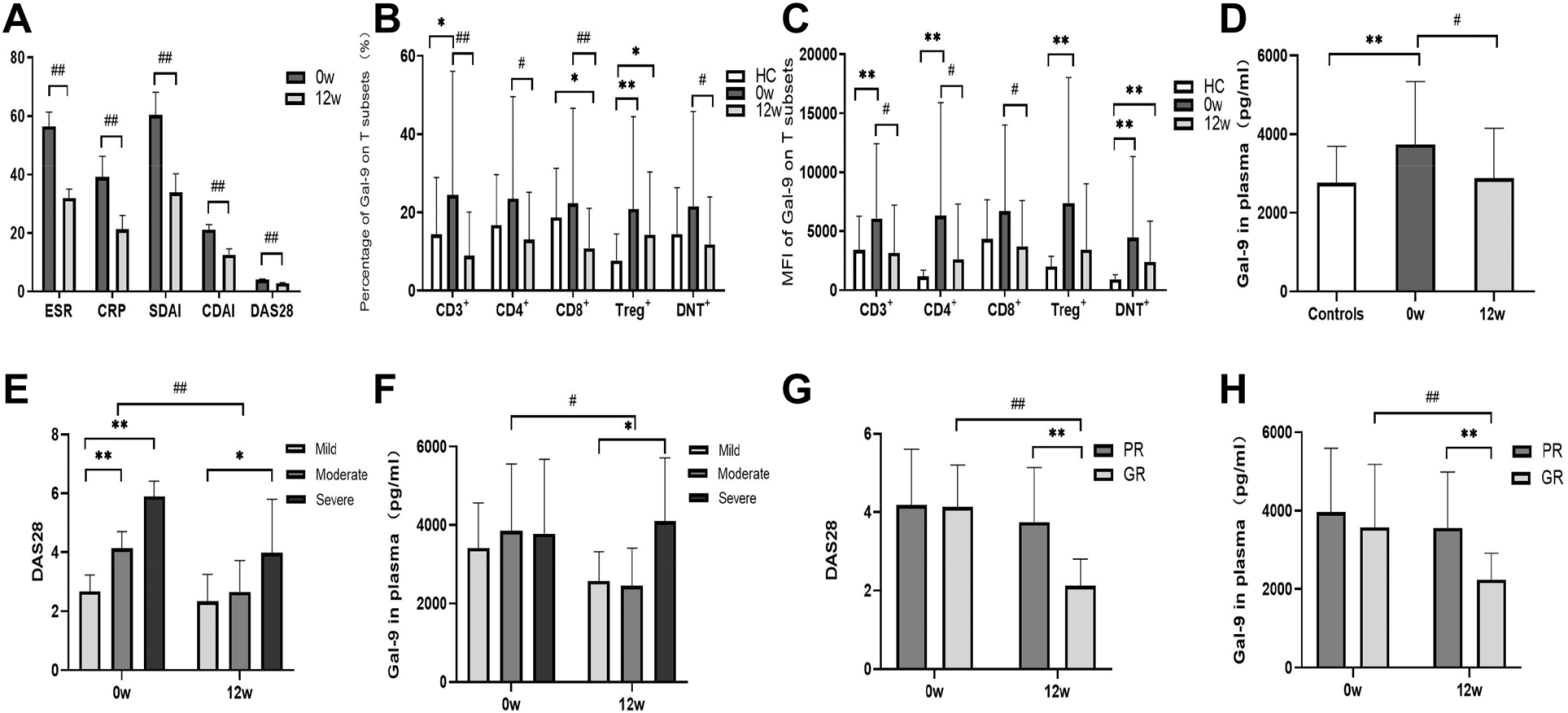
Comparison of disease activity indexes and galectin 9 (Gal-9) expression in 33 longitudinally followed rheumatoid arthritis (RA) patients at baseline (0w) and week 12 (12w). (**A**) Disease activity indexes. (**B**) Percentage of Gal-9 expression in CD3^+^, CD4^+^, CD8^+^, Treg and double-negative T cell subsets. (**C**) Gal-9 mean fluorescence intensity (MFI) in CD3^+^, CD4^+^, CD8^+^, Treg and double-negative T cell subsets. (**D**) Gal-9 levels in plasma. (**E**) Disease activity score 28 (DAS28) and (F) plasma Gal-9 levels in different disease activity groups. (**G**) DAS28 and (**H**) plasma Gal-9 levels of poor and good responders. Significant differences between different groups at the same time point are marked as *(P < 0.05) or **(P < 0.01). Significant differences between baseline and 12 weeks within the same group are marked as # (P < 0.05) or ## (P < 0.01).

The disease activity parameters ESR, CRP, T28, SW28, PGA, DAS28-CRP, SDAI and CDAI decreased significantly between baseline and week 12 after TAC-based treatment in 33 RA patients for whom pre- and post-treatment samples were available (P < 0.01; Fig. 5A). This included 1patient (3.03%) who received TAC, 5 (15.1%) who received TAC + M, 10 (30.3%) who received TAC + P, and 17 (51.5%) who received TAC + M + P. There was no significant statistical difference in Gal-9 levels between TAC + M, TAC + P and TAC + M + P groups, nor in DAS28 (Supplementary Table S2 online).

After 12 weeks of treatment, the proportion of Gal-9 expressing cells in the following T cell subsets was significantly lower than at baseline: CD3^+^T, 8.81 ± 11.04% at week 12 vs. 25.15 ± 31.9% at baseline; CD4^+^T, 12.77 ± 12.05% vs. 24.16 ± 26.29%; CD8^+^T, 10.56 ± 10.18% vs. 22.99 ± 24.41%; and double-negative cells, 11.52 ± 12.09% vs. 22.26 ± 24.37% (Fig. 5B). Mean fluorescence intensity (MFI) of anti-Gal-9 staining was significantly lower at week 12 than at baseline in CD3^+^ T (3102 ± 4004 vs. 6243 ± 6373), CD4^+^ T (2575 ± 4604 vs. 6492 ± 9701) and CD8^+^ T cells (3666 ± 3843 vs. 6854 ± 7382) (all P < 0.05), but not double-negative cells (Fig. 5C). There were no significant changes in the percentages of CD3^+^, CD4^+^, CD8^+^ or double-negative T cell subsets after TAC treatment.

The percentage of Treg cells among CD4^+^ T cells was significantly lower at week 12 (3.57 ± 1.72) than at baseline (4.54 ± 2.04, *P* = 0.041), but the percentage and MFI of Gal-9 expression in Tregs were not significantly different between the two time points.

Next, we evaluated changes in Gal-9 and cytokine levels in plasma. Baseline plasma Gal-9 levels were significantly higher in RA patients than in healthy controls (Fig. 5D). In RA patients, the concentration of plasma Gal-9 was lower at 12 weeks (2938 ± 1255 pg/ml) than at baseline (3613 ± 1569 pg/ml, *P* = 0.026; Fig. 5D). The decrease in plasma Gal-9 levels and DAS28 score between baseline and week 12 was significant in moderate RA patients but not in mild or severe patients (Fig. 5E–F). The decrease in plasma Gal-9 levels and DAS28 score between baseline and week 12 was significant in good responders but not in poor responders (Fig. 5G–H).

Both baseline and post-treatment plasma levels of VEGF, TNF-α and IL-6 were statistic higher in 33 RA patients than in controls, while these cytokines did not reduced significantly after treatment(Supplementary Fig S1 online). On the other hand, CRP level decreased significantly after treatment in 33 RA patients, which was consistent with the decline of DAS28 (Supplementary Fig S1 online).

### Comparison between treatment response groups

RA patients in the severe group had the highest disease activity at both baseline and week 12 (Fig. 5E). At week 12, plasma Gal-9 expression levels were higher in severe RA patients than in mild group (Fig. 5F).

Disease activity was lower among good responders than poor responders at week 12 (Fig. 5G). Plasma Gal-9 levels were lower in good responders (2245 ± 670 pg/ml) than in poor responders (3558 ± 1431 pg/ml, *P* = 0.006; Fig. 5H).

### Correlation of Gal-9 and other factors with disease activity

**Figure 6:**
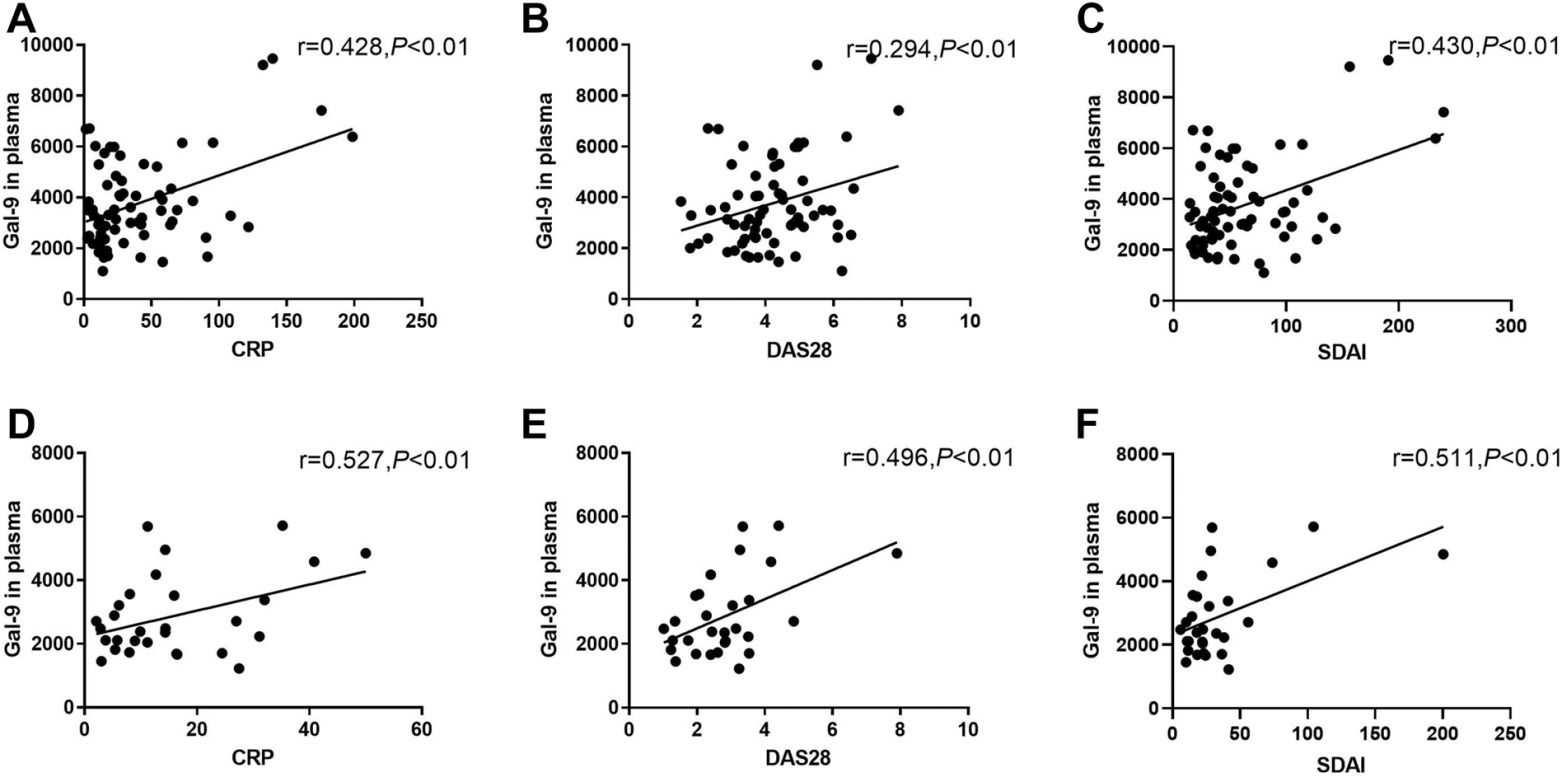
Correlation between galectin 9 (Gal-9) level in plasma and disease activity indexes in patients with rheumatoid arthritis (RA) (**A**–**C**) before treatment (n = 77) and (D-F) after treatment (n = 33). If both factors showed a normal distribution, the correlation was analyzed using the Pearson correlation test; otherwise, it was analyzed using the Spearman correlation test. **P < 0.01, *P < 0.05.

At baseline, Gal-9 levels in both PBMCs and plasma showed a moderate positive correlation with disease activity in 77 RA patients. Plasma Gal-9 levels showed a moderate positive correlation with CRP, SDAI and DAS28 in RA patients at baseline (Fig. 6A–C). Levels of CRP correlated with Gal-9 expression in CD4^+^, CD8^+^, Tregs and double-negative T cell subsets, as well as with MFI of Gal-9 expression in CD4^+^ and Treg cell subsets.

At week 12, plasma Gal-9 levels in 33 patients post-treatment showed a moderate positive correlation with CRP, DAS28, and SDAI (Fig. 6D–F). However, neither the percentage nor MFI of Gal-9 expression in T cell subsets correlated with disease activity in the 33 patients. Taken together, these results indicate that plasma Gal-9 levels showed a strongest positive correlation with disease activity than percentage and MFI of Gal-9 expression in T cell subsets.

There is no significant difference in correlations between Gal-9 and other three cytokines such as VEGF, TNF-α, and IL-6 in plasma at baseline and after treatment. There is also no correlation between these three cytokines (Supplementary Table S3 online).

## Discussion

In our prospective cohort study, we evaluated the features of Gal-9 as a biological marker for drug response, as well as its relationship with disease activity in active RA patients. Gal-9 expression in several T cell subsets and plasma of RA patients was significantly higher than in healthy controls, consistent with our previous observations^15^. Moreover, our data showed that, after TAC treatment for 12 weeks, Gal-9 expression level in good responders was significantly lower than in poor responders. The data on DAS28 and Gal-9 levels in the different treatment groups indicated that the combination of MTX and Pred had no additive effect on TAC treatment. TAC presumably acted as a strong immunosuppressant, and the core determine on RA remission and declining in Gal-9 levels. Additionally, Gal-9 expression level in plasma was positively correlated with DAS28 score both at baseline and at week 12 post-treatment.

Our results indicated that Gal-9 might play an important role in the immunoregulation of RA and can provide efficacy and prognostic information in RA patients, accurately reflecting patient response to TAC-based treatment. Gal-9 could be regarded as a sensitive indicator to monitor response to TAC or other DMARDs. The reason that Gal-9 potentiates the immunoregulatory effects of TAC therapy on RA patients was still elusive, and would be explored in future. In respect of its immunomodulatory functions, Gal-9 was initially described as a negative regulator of T cell immunity by inducing apoptotic cell death in CD4^+^ T helper 1 (Th1) and T helper 17 cells^16^. Lee et al. found that the mRNA levels of Gal-9 were higher in PBMC of RA patients than healthy individuals, and higher in patients with low disease activity than those with moderate to high disease activity^17^. Percentage of Gal-9^+^ cells in synovial tissue and the concentration of Gal-9 in the synovial fluid of RA patients increased remarkably than OA patients, and negatively related to disease activity in RA patients^18^. Current researches also showed up high-dose Gal-9 treatment alleviated inflammation response in mouse models of autoimmune diseases^16,19^.

Recent study that serum levels of Gal-9 were increased in RA patients and associated with disease activity in RA patients without high titers of ACPA were published, which suggested that Gal-9 possessed the properties of pro-inflammatory or arthropathic biomarker^20^. We tracked the ACPA level of 19 patients in this study and analysis the relationship between Gal-9 levels and ACPA titer, there were no confirm correlation among ACPA antibody and Gal-9 levels at baseline and 12 week (Supplementary Table S4 online). Fujita Y found hat patients in remission had high levels of galectin-9 compared to the moderate activity group^21^. The results above are mainly based on cross-sectional study. Our prospective study not only indicates higher expression of Gal-9 in PBMC and plasma in RA than healthy controls, which is in consistent with previous studies^10,22,23^, but also proved that Gal-9 levels decrease along with remission of disease activity and had positive relation with disease activity index. Taken together,it demonstrated that Gal-9 might act as a candidate index for RA disease activity, analogous to the way in which serum levels of Gal-9 were significantly elevated in patients with systemic lupus erythematosus and correlate with disease activity^24^. Gal-9 and C-X-C motif chemokine 10 might be reliable biomarkers for disease activity in juvenile dermatomyositis^25^.

The functions of Gal-9 are complex and even conflicting according to the literature. This might be explained by the location that binds to glycosylated proteins, the diverse properties of its isoforms, the unique structures of the N- and C-terminal carbohydrate recognition domains, and its different receptors, such as Tim-3 on immunocytes^26^. Seki et al. found that knockdown of Gal-9 by small interference RNA induced apoptosis in the synovium of RA, which suggested that endogenous and exogenous forms of Gal-9 played opposing roles in regulating cell death^27^. Previous work demonstrated that low concentrations (0.5 µg/ml) of Gal-9 activate and expand IFN-γ-producing CD4^+^ Th1 cells^28,29^. Our previous study indicated there were significantly higher inflammation and monocyte migration when C57BL/6 mouse knees were directly injected with Gal-9 medium (1.39 µmol/l) than negative controls^30^. Therefore, Gal-9 might exert opposite effects on immune regulation and inflammatory responses, depending on whether it is present at physiological levels or overexpressed. This should be investigated in further study.

Gal-9 is also expressed in vascular endothelial cells with several functions, including modulating the phosphorylation of VEGF-receptor3 and insulin-like growth factor 1, regulating the RTK pathway, mediating the adhesion between tumoral and endothelial cells, regulating the proliferation and metastasis of tumor cells^31^. Rodriguez-Carrio J et al. reported reduced numbers of angiogenic T cells and endothelial progenitor cells in RA patients^32^, but another study found an elevated percentage of circulating angiogenic T cells that positively correlated with the percentage of endothelial progenitor cells in RA patients, which regulated VEGF levels through Akt signaling with CD147^33^. Our previous cross-sectional study on Gal-9 level in 105 RA patients have included the same baseline data of some RA patients in this prospective cohort^15^. In the current study, the concentrations of Gal-9 and VEGF in RA patients' plasma decreased after TAC-based treatment. However, we could not find a correlation between Gal-9 and VEGF in plasma, probably due to the relatively small number of available samples. Further studies are needed on the relationship between Gal-9 and VEGF in angiogenesis and regulation of inflammation in RA.

Our research presents some limitations. First, we conducted a prospective cohort study with limited longitudinal data, since the withdrawal rate and percentage of patients with missing samples were high. However, the baseline clinical data and demographic characteristics of patients in each observed population were consistent with the full study cohort of 96 RA patients. It revealed that lost cases did not lead to bias. Second, we were unable to determine which T cell subsets were key in regulating Gal-9 expression, or analyze the interaction and feedback among the subsets. Last, with the flaw of this one-arm prospective study was the lacking data of those RA controls who receive any cDMARDs other than TAC, We supposed that Gal-9 might play an important role in the immunoregulation of RA and can provide efficacy and prognostic information in RA patients, reflecting patient response to TAC-based treatment. Further research on these questions is required.

## Conclusions

Gal-9 was positively related with disease activity in RA patients. Lower Gal-9 levels at week 12 were associated with better response to TAC-based treatment. Gal-9 may be useful as a new biomarker for evaluating RA activity and therapeutic effect.

## Materials and methods

### Study cohort

This one-armed prospective study involved 96 RA patients in the outpatient clinics and wards of the rheumatology department in Qilu Hospital of Shandong University from January 2015 to December 2017. Patients with following conditions were included in this study: patients who met the ACR diagnostic criteria in 1987 were enrolled in this study, whether their ACPA/RF positive or not; older than 18 years; stable extra-articular manifestations; be intolerant or unresponsive to three month’s cDMARD, glucocorticoid, biological agents and/or traditional Chinese medicine^7^. The exclusion criteria were: acute or chronic infection (bacteria, fungi and virus including tuberculosis or hepatitis); severely abnormal blood cell counts (white blood cells < 3 × 10^9^/L, platelets < 80 × 10^9^/L); glutamic-pyruvic transaminase or glutamic-oxalacetic transaminase greater than two times the normal upper limit; renal insufficiency; pregnant or lactating women; history of malignant tumors; severe hypertension, diabetes or coronary heart disease or other autoimmune diseases.

Patients’ medication history and clinical data were collected at baseline and after treatment for 12 weeks. All patients in our study received low-dose TAC (2 mg/day) at the onset of the treatment, and they were treated with TAC monotherapy or TAC combined with methotrexate (MTX, M) (TAC + M) or/and prednisone (Pred, P) (TAC + P/TAC + M + P) for 12 weeks based on previous medication, disease activity, age and external articular manifestations. At the same time, 58 healthy control individuals were recruited from the physical examination center of Qilu Hospital of Shandong University, and the gender composition and age of controls were matched with the RA patients. Whole blood was sampled in ethylenediaminetetraacetic acid (EDTA) anticoagulant.

The study was approved by the Human Research Ethics Committees of Qilu Hospital of Shandong University (KYLL-2015-269) from January 2015 to December 2018. All methods were carried out in accordance with relevant guidelines and regulations. Written informed consents were obtained from all participants. Clinical trials was registered on *ClinicalTrials.gov* (NCT02837978, registered in 20/07/2016).

### Clinical trial flow and patient enrolling

A total of 96 RA patients were included in the study, for 77 blood samples at baseline were available. 50 of 77 completed the 12-week clinical follow-up, and post-treatment blood samples were available for 33. Unfortunately, baseline blood samples of 19 RA patients weren’t tested for variety reasons. However, they still accepted the TAC treatment and finished regular follow-up, with their blood samples were collected at week 12. Thus, there were a total of 52 patients with immune data at week 12 after therapy. Among 96 enrolled RA patients, 18 cases stopped tacrolimus treatment due to financial issue, adverse events, inefficiency and intolerance reason, and 9 cases were lost with unknown. We didn’t continue to follow-up the disease activity and Gal-9 levels of these patients. The flowchart of patient enrollingwas shown in Fig. 1.

At baseline, the 77 RA patients were divided into a mild group, defined as those showing a disease activity score ≤ 3.2 on the Disease Activity Score in 28 joints (DAS28) score (n = 16); moderate group, defined as those with a disease activity score > 3.2 and ≤ 5.1 (n = 44); and severe group, defined as those with a score > 5.1 (n = 17). Patients were divided into two groups (CDAI > 10, n = 70 and CDAI ≤ 10, n = 70) depending on their clinical disease activity index (CDAI)^34^.

If the DAS28 score of patients was lower than 3.2 and decreased more than 0.6 after treatment, the subject was classified as good responser to treatment (GR group, GR); otherwise, he or she was defined as poor responser (PR group, PR)^35^.

We made statistic on the four treatment methods (TAC, TAC + MTX, TAC + Pred, TAC + MTX + Pred) of 33 RA patients who completed the follow-up. There was only one patient with TAC monotherapy. Therefore, we analyzed the changes of DAS28 and Gal-9 levels in the other three treatment subgroups at baseline and 12 week (Supplementary Table S2 online).

The disease activity parameters including Erythrocyte sedimentation rate(ESR) , C-reactive protein(CRP), Tender joints of 28 counted(T28), Swollen joints of 28 counted(SW28), Patient global assessment(PGA), Physician global assessment(PHGA), Health Assessment Questionnaire(HAQ), Simplified disease activity index(SDAI), CDAI, DAS28 were recorded for all RA patients.

### Immunodetection methods

PBMCs were separated from whole blood samples by density gradient centrifugation. Flow cytometry was performed on PBMCs of RA patients and controls. Antibodies CD3-APC-eFluor780, CD8-FITC, CD127-PE-Cyanine7 (eBioscience, San Diego, CA, USA), CD4-Alexa Fluor 700, and CD25-PE/Dazzle 594 (BioLegend, San Diego, CA, USA) were used for extracellular identification of the different T cell subsets. The staining procedure was as follows: extracellular staining and incubation were performed for 20 min, then 100 μl of IC Fixation Buffer (eBioscience) were added and cells were incubated for 20 min. Cells were washed and 100 μl of Perm Wash Buffer (eBioscience) were added. Subsequently, 5 μl of antibody anti-Gal-9-PE (BioLegend) were added, cells were incubated for 20 min at room temperature, and the supernatant was discarded after washing. Phosphate-buffered saline (PBS) was added to resuspend the cells, and the solution was filtered. Flow cytometry was performed (BD Biosciences, Beckman Navios, Franklin Lakes, NJ, USA) within 24 h. The gating strategy of T cell subsets in peripheral blood mononuclear cells (PBMCs) of one rheumatoid arthritis patient in flow cytometry analysis were shown in Fig. 2. Data were analyzed using FlowJo software version 7.6 (Tree Star, Ashland, OR, USA).

The concentrations of vascular endothelial growth factor (VEGF), TNF-α and IL-6 in plasma were determined by ELISA kits (eBioscience, San Diego, CA, USA), the Gal-9 ELISA from R&D Systems (MN, USA) according to the manufacturer’s protocol.

### Statstical methods

SPSS 22.0 software (IBM, Armonk, NY, USA) was used for statistical analysis. All data of RA patients and controls at baseline and at week 12 were tested for normal distribution using the homogeneity of variance test. If variance was homogeneous, the independent-samples t-test was used; otherwise, the t’-test was used for comparison between two groups. One-way ANOVA and post hoc comparison with Fisher’s least significant test were used for multiple groups. A paired t-test was used to analyze data from the 33 RA patients with samples available before and after treatment.

A correlation analysis was conducted on each indicator for 77 patients at baseline, and 33 patients with RA that were longitudinally followed and with samples available at baseline and week 12. The Pearson correlation test was applied to normally distributed data; the Spearman's test, to skewed data. Data were reported as mean ± standard deviation (SD).

## Supplementary Information

Supplementary Information
