# Supplementary material for: Galectin-9 expression correlates with therapeutic effect in rheumatoid arthritis
Source: Sci Rep. 2021 Mar 10;11:5562. doi: 10.1038/s41598-021-85152-2 (PMC7946964; doi:10.1038/s41598-021-85152-2)

Galectin-9 expression correlates with therapeutic effect in rheumatoid arthritis

Jiao Sun[1,2], Yameng Sui[1,3], Yunqing Wang[1,4], Lijun Song[1,5], Dong Li[1,6], Guosheng Li[1,7], Jianwei Liu[1], Qiang Shu[1,5]*

*Correspondence: shuqiang@sdu.edu.cn

[1] Department of Rheumatology, Qilu Hospital of Shandong University, Ji'nan 250012, Shandong, China.

[2] Department of Nephrology and Immunology, Shandong Provincial Third Hospital, Shandong University, Ji'nan 250031, Shandong, China

[3] Department of Rheumatology and Immunology, Yantai Mountain Hospital, Yantai 264001, Shandong, China.

[4] Department of Rheumatology and Immunology, Guangdong Second Provincial General Hospital, Guangzhou 510317, Guangdong, China.

[5] Shenzhen Research Institute of Shandong University, Shenzhen 518057, Guangdong, China.

[6] Cryomedicine Laboratory, Qilu Hospital of Shandong University, Ji'nan 250012, Shandong, China.

[7] Department of Hematology, Qilu Hospital of Shandong University, Ji'nan 250012, Shandong, China

Table S1. Dosage of TAC, MTX, and Pred in different disease activity groups

		Mild group (n=16)	Moderate group (n=44)	Severe group (n=17)
DAS28,(X±SD)	0w	2.49±0.58	4.15±0.57**	5.96±0.7**
Dosage of TAC(mg/d), median(IQR)	0w	2(1,2)	2(2,2)	2(1,2)
	12w	2(1,2.5)	2(0.75,2)	2(1,2)
Dosage of MTX(mg/w) , median(IQR)	0w	10(7.5,10)	10(5,12.5)	10(5,15)
	12w	10(7.5,15)	10(5,15)	10(7.5,10)
Dosage of Pred(mg/d), median(IQR)	0w	8.75(2.5,20)	10(5,30)	15(5,50)
	12w	7.5(2.5,17.5)	10(2.5,15)	10(5,15)
Concentration of TAC(ng/ml), median(IQR)	3w	3(2,10.7) （n=6）	4.8(1.8,14.5) （n=15）	6.6(5.5,26) （n=3）

DAS28: Disease Activity Score-28; TAC: tacrolimus; MTX: methotrexate; Pred: prednisolone; IQR: interquartile range; The drug dose between three groups was tested by non-parametric test, while DAS28 was analyzed by ANOVA. Statistical difference of different groups at same time point was marked compared to mild group by*, *P<0.05, **P<0.01.

Table S2. Changes of Galectin-9 levels in different treatment subgroups from baseline to 12weeks

	T+M+P (n=17)		T+M (n=10)		T+P (n=5)	
	0w	12w	0w	12w	0w	12w
DAS28	4.52±1.09	2.88±0.96##	3.95±1.51	3.1±1.92#	3.3±0.78	2.06±0.89
pGal-9(pg/ml)	3313±1684	2461±1140	4396±1403	3343±1407	4284±1193	2694±1056
$CD3^+Gal\text{-}9^+$(%)	21.87±27.81	8.57±14.05	21.56±34.36	9.3±8.63	31.54±39.07	7.48±5.09
$CD4^+Ga\text{-}9^+$(%)	21.18±23.24	8.89±7.96	29.66±37.51	18.28±13.71	17.78±13.2	12.84±17.8
$CD8^+Gal\text{-}9^+$(%)	19.88±22.06	8.26±7.27	28.81±35.6	12.65±11.25	20.76±7.98	13.41±16.64
$Treg^+Gal\text{-}9^+$(%)	15.74±14.12	9.89±10.83	27.32±33.19	16.58±17.44	15.05±19.05	14.66±20.72
$DNTGal\text{-}9^+$(%)	22.22±21.15	7.72±7.58	27.73±35.8	15.07±14.45	8.26±4.23	17.06±17.84

DAS28: Disease Activity Score-28; Gal-9: Galectin-9; T: Tacrolimus; M: MTX; P: Prednisone; Treg+: regulatory T cells, DNT+: Double negative T cell, T+Gal+(%): percentage of T cell expressed Gal-9. pGal9 and DAS28 was analyzed by ANOVA, while other different index was tested by non-parametric test. Statistical difference between different subgroups at same time point was marked by*, *P<0.05. Self-control analysis between 0w and 12w of same subgroup was marked by#, #P<0.05, ##P<0.01; Values are shown as X±SD.

Table S3. The correlations of pGal-9 with different cytokine levels in RA patients at baseline

	pGal-9 (pg/ml)	pVEGF (pg/ml)	pTNF-α(pg/ml)
pVEGF(pg/ml)	-0.06		
pTNF-α(pg/ml)	0.10	0.21	
pIL-6(pg/ml)	0.23	0.25	0.41

The correlation was analyzed using the Spearman correlation test. **P<0.01, *P<0.05.

Table S4. Gal-9 levels in different self-antibody subgroup of RA patients

		ACPA		
		Negative (n=6)	Low titers of ACPA titers (< 200 U/ml) (n=3)	High titers of ACPA titers (> 200 U/ml) (n=10)
DAS28-CRP	0w	3.49±0.54	2.85±0.48	4.11±1.31
	12w	2.3±0.54	3.17±1.63	2.77±1.63
pGal-9(pg/ml)	0w	3930±1999	4376±1426	3307±2001
	12w	2825±85	3052±959	2843±522
$CD3^+Gal^+$(%)	0w	8.91±4.09	13.18±10.23	39.29±38.29
	12w	11.36±10.17	10.67±12.64	29.46±41.64
$CD4^+Gal^+$(%)	0w	18.19±11.68	8.04±5.64	16.25±11.66
	12w	11.58±5.02	17.81±11.85	11.41±16.11
$CD8^+Gal^+$(%)	0w	22.83±6.89	16.65±12.89	19.38±8.72
	12w	10.2±7.54	17.95±16.43	8.66±12.22
$Treg^+Gal^+$(%)	0w	23.92±22.2	18.37±22.63	20.79±17.11
	12w	7.55±5.75	22.07±21.27	14.17±19.56
$DNTGal^+$(%)	0w	15.18±10.18	17.21±20.02	18.08±9.95
	12w	19.61±19.5	17.08±12.09	6.95±9.83

Gal-9: Galectin-9; ACPA: Anti-citrullinated peptide antibody, RF: rheumatoid factors; $T^+Gal\text{-}9^+$(%): percentage of T cell expressed Gal-9. pGal9 and DAS28 was analyzed by ANOVA, while other

different index was tested by non-parametric test. Statistical difference between different groups at same time point was marked by*, **P*<0.05, ***P*<0.01. Values are shown as X±SD.

Figure S1. VEGF, TNF-α, IL-6 and CRP expression in 33 RA patients at baseline and after treatment

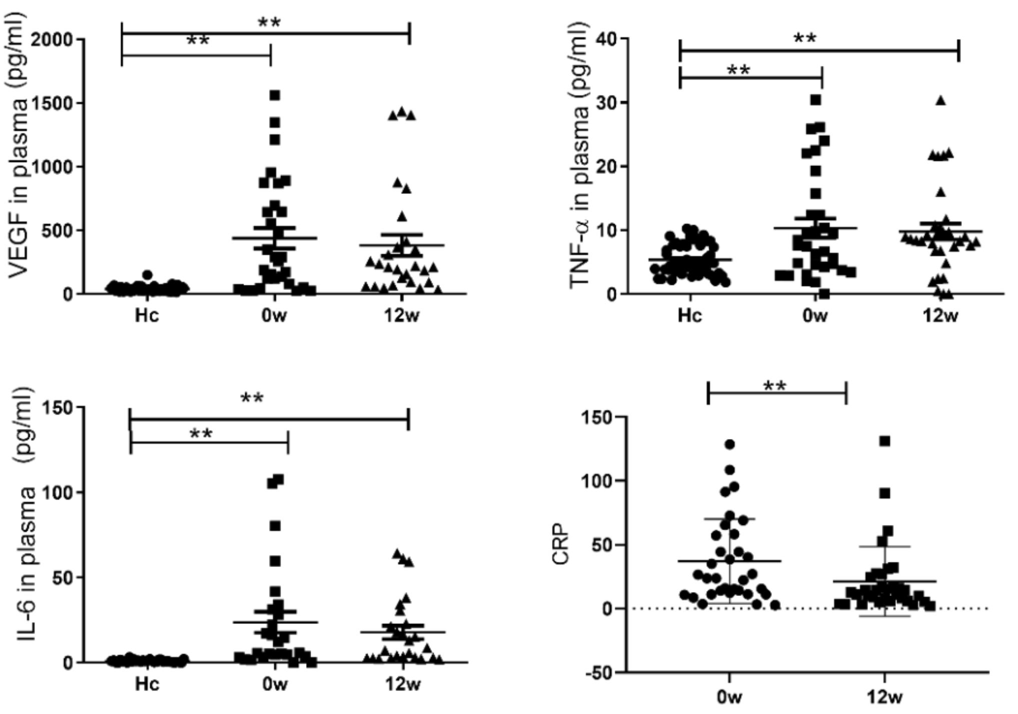

Supplement: Supplementary file 1 — Supplementary Information [file 41598_2021_85152_MOESM1_ESM.pdf]
